# Necrotizing Pneumonia Mimicking Lung Tumour: A Case Report

**DOI:** 10.1002/rcr2.70264

**Published:** 2025-07-08

**Authors:** Rahel Yuana Sadikim, Isnin Anang Marhana, Herley Windo Setiawan

**Affiliations:** ^1^ Department of Pulmonology and Respiratory Medicine, Faculty of Medicine Airlangga University Surabaya Indonesia; ^2^ Department of Pulmonology and Respiratory Medicine Dr. Soetomo General Academic Hospital Surabaya Indonesia

**Keywords:** community‐acquired infections, diagnostic imaging, *Klebsiella pneumoniae*, necrotizing pneumonia

## Abstract

Necrotizing pneumonia, a rare but severe complication of community‐acquired pneumonia, can mimic lung tumours. This case report highlights the importance of early diagnosis using contrast‐enhanced chest CT scans and a multidisciplinary approach to prevent misdiagnosis that can increase morbidity and mortality. A 57‐year‐old male presented with severe respiratory symptoms, including haemoptysis and weight loss. The patient was initially misdiagnosed with lung cancer due to a necrotic mass based on imaging. Subsequent biopsies revealed no signs of malignancy. A multidisciplinary team concluded that the findings suggested necrotizing pneumonia rather than a lung tumour. The patient was successfully treated with antibiotics, leading to significant improvement. This case highlighted the importance of accurate and timely diagnosis in managing necrotizing pneumonia, particularly when it mimics malignancy. Early recognition, supported by appropriate diagnostic tools and targeted antibiotic therapy, is crucial for appropriate management of necrotizing pneumonia and achieving positive outcomes. A multidisciplinary approach is essential to ensure comprehensive care, prevent misdiagnosis and avoid unnecessary treatments. Prompt and precise intervention can significantly enhance patient prognosis and reduce the risk of complications associated with necrotizing pneumonia.

## Graphical abstract

This case report describes a male patient with necrotizing pneumonia mimicking a lung tumour and highlights the importance of accurate and timely diagnosis in managing necrotizing pneumonia, particularly when it mimics malignancy.

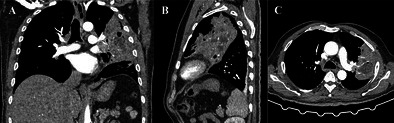

## 1 Introduction

Necrotizing pneumonia is a severe form of community‐acquired pneumonia [1]. This condition was first identified in adults in the 1940s and in children around 50 years later. Epidemiologically, the incidence of necrotizing pneumonia in the United States is 0.8%–7% in children and less than 1% in adults [2]. No cases have been reported in Asia, likely due to limited research and rare case reports [3].

The most common pathogens causing necrotizing pneumonia include *Streptococcus pneumoniae, S. aureus* and *Klebsiella pneumoniae* [2]. Clinically, necrotizing pneumonia resembles severe pneumonia, with symptoms including cough, haemoptysis, fever, shortness of breath and chest pain [2, 3]. Contrast‐enhanced chest CT scans are considered the most sensitive diagnostic modality, showing patchy or diffuse consolidation, multiple thin‐walled small cavities (less than 1 cm) without peripheral contrast enhancement at the cavity, and peripheral necrosis areas [2, 4, 5]. Delayed treatment significantly increases morbidity and mortality [2]. This case report describes a male patient with necrotizing pneumonia mimicking a lung tumour.

## 2 Case Report

A 57‐year‐old male presented to the emergency department with a 5‐month history of chronic intermittent high fever, productive cough and shortness of breath. These symptoms had worsened significantly over the preceding 5 days. The patient also experienced chronic intermittent haemoptysis, having escalated to 20–30 mL/day in the past 2 days. Additional symptoms included sharp left‐sided chest pain radiating to the back and an unintentional weight loss of 10 kg over the previous 4 months. The patient had a 3‐year history of type 2 diabetes, managed with Metformin.

Initially, the patient was clinically diagnosed with tuberculosis (TB) based on a chest x‐ray, despite a negative sputum test for *Mycobacterium tuberculosis* . He was started on anti‐tubercular therapy (ATT). However, due to worsening haemoptysis and continued weight loss, ATT was discontinued after 20 days. The patient had been hospitalised twice: first for haemoptysis, during which a chest CT scan and biopsy were performed, and second for bronchoscopy. GeneXpert analysis of bronchoalveolar lavage (BAL) samples yielded negative results.

There was no family history of cancer or tuberculosis. The patient had a 20‐year history of smoking one pack of cigarettes per day, which he had quit smoking 12 years prior. He denied alcohol and drug abuse. No previous history of TB exposure was known.

Upon admission, the vital signs of patient were blood pressure (117/72 mmHg), pulse rate (71 beats per minute) and temperature (38.7°C). The patient was tachypneic with a respiratory rate of 26 breaths/min and a SpO2 of 98% with a simple mask at 6 L/min. The Visual Analogue Scale (VAS) score was between 3 and 4 which indicates mild to moderate pain, with sharp left‐sided chest pain radiating to the back and is intermittent. Physical examination revealed anaemia, decreased vesicular sounds and crackles in the upper two‐thirds of the left hemithorax. Laboratory examinations revealed a haemoglobin level of 9 g/dL with a mean corpuscular volume (MCV) of 72.6 fL and mean corpuscular haemoglobin (MCH) of 23.9 pg. The white blood cell count was at 19,590 cells/μL with 86% neutrophils. Additional findings included albumin (2.86 g/dL), C‐reactive protein (44.86 mg/L, approximately 44 times higher than the upper normal limit), lactate dehydrogenase (252 U/L), carcinoembryonic antigen (CEA) (8.94 ng/mL) and uncompensated respiratory acidosis with hypoxemia.

**FIGURE 1:**
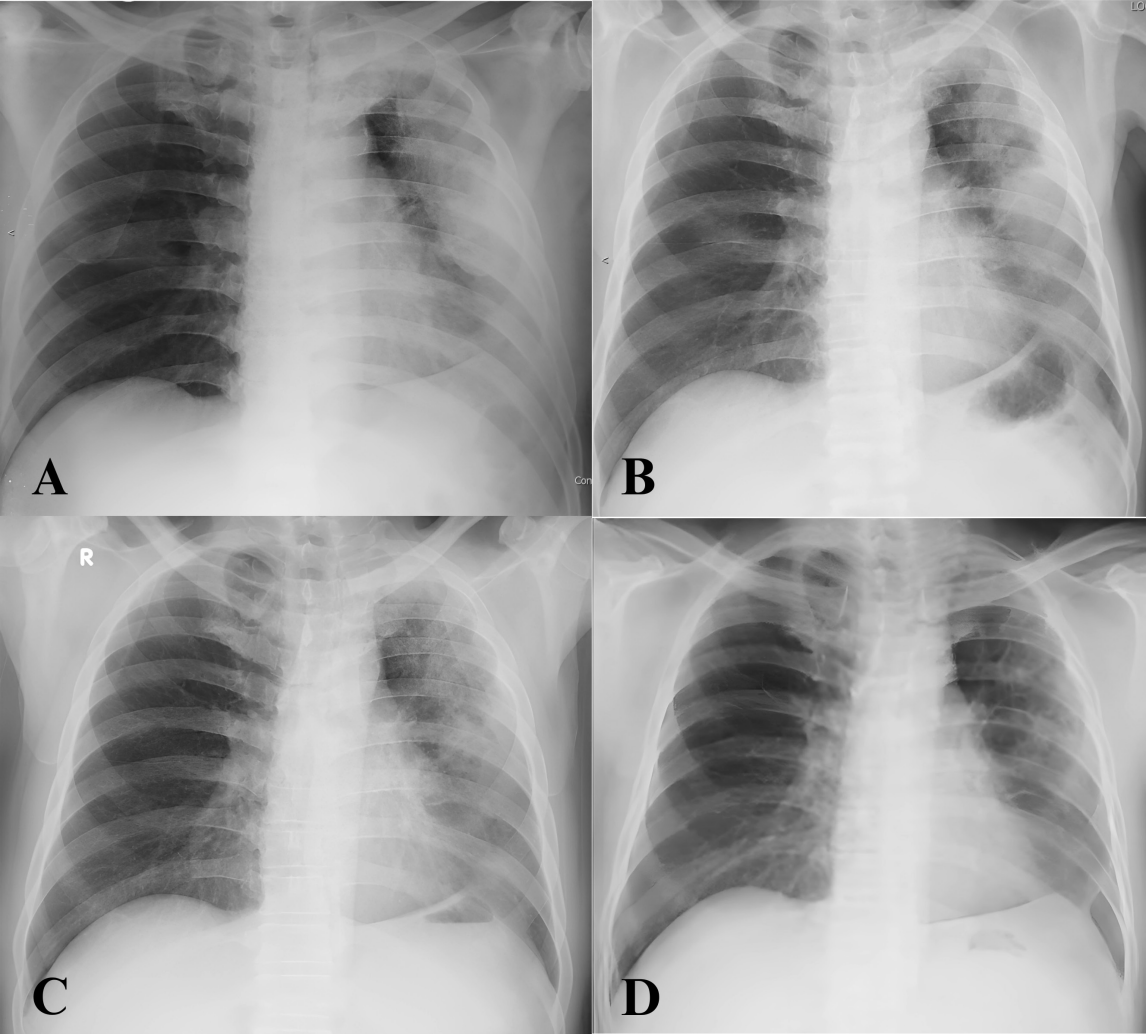
Chest x‐ray findings. Day‐1 admission (A), day‐5 admission (B), day‐15 after receiving an oral antibiotic for 14 days in total (C) and follow‐up 2 months after treatment (D).

**FIGURE 2:**
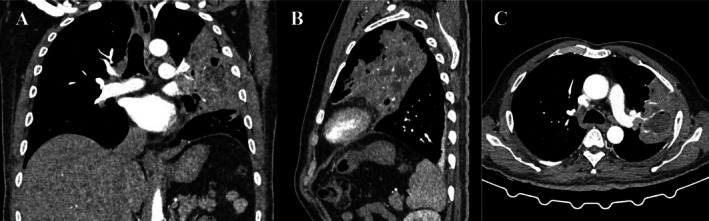
Computed tomography findings. Coronal (A), sagittal (B) and axial (C) planes. A second opinion contrast‐enhanced CT scan revealed an area of consolidation with multiple small cavities, necrotic component (24 HU) and contrast enhancement (79 HU) of the upper left lobe, indicating necrotizing pneumonia with mucous plaque.

**FIGURE 3:**
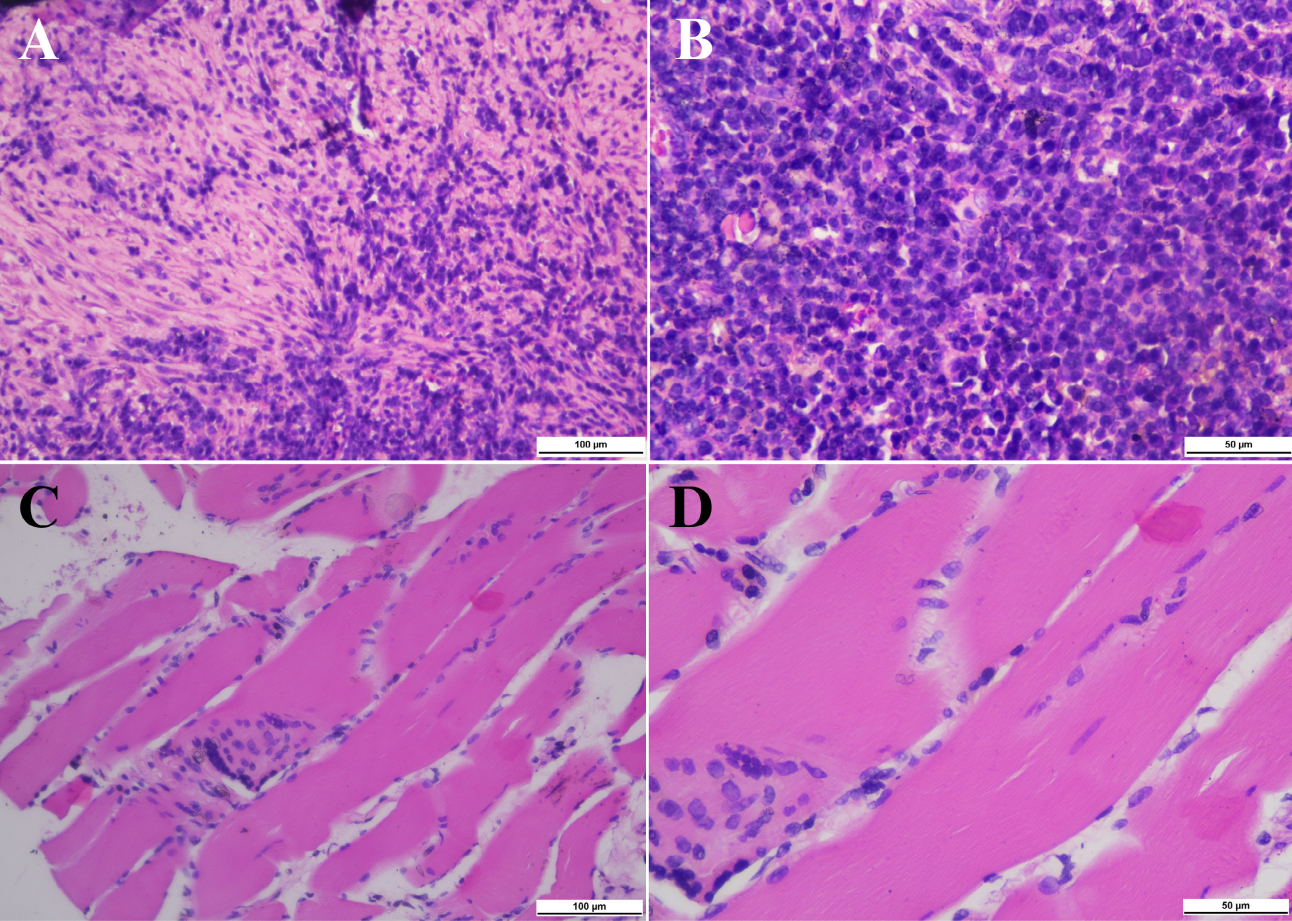
CT‐guided core biopsy. (A, B) H&E staining revealed tissue section without epithelial lining with inflammatory cell debris including lymphocytes, histiocytes, neutrophils and plasma cells. (C, D) H&E staining revealed tissue composed of muscle. The muscle component referred to in the figure corresponds to a section of skeletal muscle tissue, characterised by striated fibres and peripherally located nuclei. This tissue was likely obtained during the core biopsy procedure. These findings showed chronic suppurative inflammation (A, C: ×200 and B, D: ×400).

**FIGURE 4:**
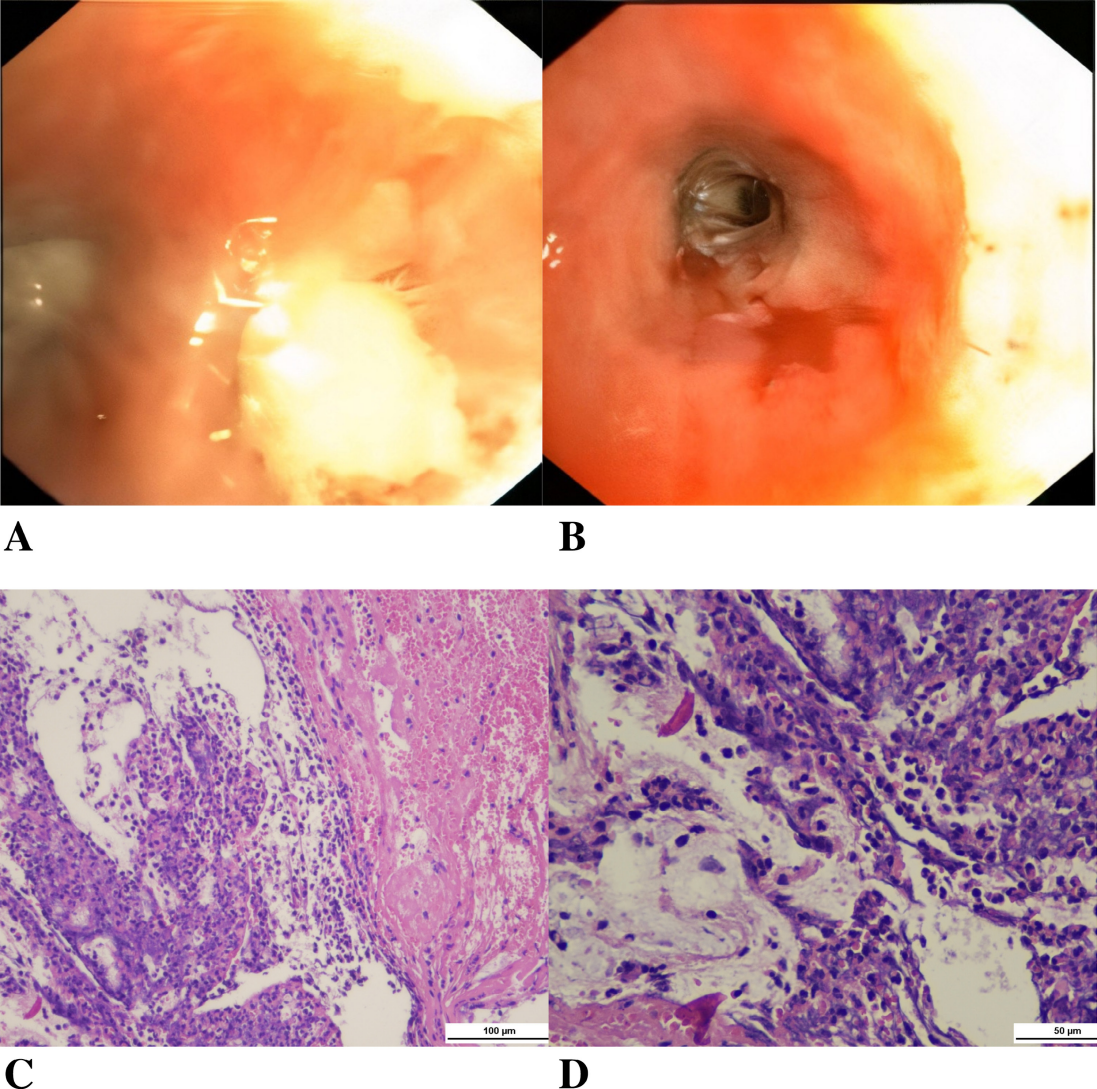
Bronchoscopy findings. (A, B) Mucosal plaque in the upper division of left superior lobar bronchus and the lingula bronchus, irregular and easily bleeding mucosa in the lingula, with no intraluminal mass or compression (A: Upper division of left superior lobar bronchus, B: The lingula bronchus). (C, D) H&E staining revealed alveolar tissue with inflammatory cell debris including lymphocytes and histiocytes, along with haemorrhagic areas (C: ×200 and D: ×400).

A chest x‐ray demonstrated a mass opacity with indistinct borders and a pneumonic reaction in the left upper lobe (Figure 1A). A contrast‐enhanced CT scan at a previous hospital revealed a solid mass measuring 9.4 × 5.7 × 10.3 cm with necrotic components in the left upper lobe (Figure 2A–C). These findings were suggestive of a malignant lung mass. However, a CT‐guided core biopsy (Figure 3A,B) and bronchoscopy (Figure 4A–D) performed at the previous hospital showed no evidence of malignancy.

The patient was admitted to our hospital and initial diagnosed with community‐acquired pneumonia, a left lung tumour, grade 2 haemoptysis, hypochromic microcytic anaemia, hypoalbuminemia and normoglycemic type 2 diabetes mellitus. He received supplemental oxygen therapy and intravenous empirical antimicrobial treatment with cefoperazone‐sulbactam. Additional supportive treatments included packed red blood cell transfusions to maintain haemoglobin levels at or above 10 g/dL, intravenous tranexamic acid, intravenous vitamin K, oral codeine and subcutaneous insulin.

By the fifth day of hospitalisation, the patient showed clinical improvement, with normalised C‐reactive protein levels (0.26 mg/L) and reduced heterogeneous opacity in the left lung (Figure 1B). Aerobic sputum cultures identified non‐ESBL‐producing *K. pneumoniae* , resistant to ampicillin, chloramphenicol and piperacillin. Intravenous cefoperazone‐sulbactam was administered for 7 days. A second CT‐guided core biopsy was performed, which again revealed no malignant cells (Figure 3C,D). On the seventh day of hospitalisation, a multidisciplinary team, comprising radiologists, pathologists, thoracic surgeons and pulmonologists from the infection and oncology divisions, reviewed prior contrast‐enhanced CT scans and the pathological findings from the third biopsy. A second‐opinion contrast‐enhanced CT scan was performed (Figure 2A–C) and confirmed the diagnosis of necrotizing pneumonia rather than a lung tumour.

The patient was deemed fit for discharge, and antibiotic treatment was transitioned to oral amoxicillin‐clavulanate, based on the sensitivity of aerobic sputum culture, for 14 days in total. Follow‐up was performed through clinical assessments and chest x‐rays (Figure 1C,D). The patient showed significant clinical and radiological improvement, with a body weight gain of 7.5 kg over 2 months.

## 3 Discussion

Community‐acquired pneumonia (CAP) remains a leading cause of hospital admissions, with 3.7%–12% of cases progressing to necrotizing pneumonia (NP) [6]. NP is marked by the rapid progression of pulmonary inflammation, characterised by consolidation, cavitation and peripheral necrosis of pulmonary tissue [1]. Retrospective studies have shown an increasing NP incidence over the past two decades in regions such as the United Kingdom, Europe, Australia and Israel. This rise is likely associated with enhanced clinician awareness, improved utilisation of contrast‐enhanced chest CT scans, evolving bacterial patterns and antibiotic use [6, 7].

The pathophysiology of NP is complex and not yet fully understood. Cytokine‐mediated inflammatory responses to pathogen invasion play a crucial role. Recent studies reveal that abnormal coagulation, vascular occlusion and venous thrombosis due to vasculitis are also central to NP development, potentially due to decreased perfusion [3, 4]. Different pathogens contribute to NP through varying mechanisms [8]. The causative pathogens of NP are diverse, ranging from typical and atypical bacteria to anaerobes, *M. tuberculosis* , fungi and viruses. Among these, *Staphylococcus aureus, S. pneumoniae, K. pneumoniae* and *Mycoplasma pneumoniae* are the most prevalent bacterial causes in adults [2, 3]. However, the causative organisms can be identified in fewer than half of the cases (approximately 11% in several studies), possibly due to early antibiotic administration prior to the onset of severe symptoms [6].

Necrotizing pneumonia typically develops over several days and can lead to serious complications such as respiratory failure, sepsis, septic shock and multi‐organ dysfunction [2, 9]. Risk factors associated with NP include immunocompromised states, hospitalisation, high‐risk aspiration, diabetes mellitus, chronic liver disease, malignancy, alcohol abuse, a history of smoking and corticosteroid therapy [8, 9]. In the present case, the patient had a history of diabetes mellitus, which is frequently associated with NP.

Common clinical symptoms of NP include cough, haemoptysis, fever, dyspnea, chest pain and weight loss in patients with more indolent infections [8]. Physical examination often reveals tachypnea, signs of consolidation and additional breath sounds such as wheezing and crackles [3, 6]. Laboratory findings typically include leukocytosis, neutrophilia, lymphopenia and elevated inflammatory markers [2]. Adequate sputum samples are essential for Gram staining and aerobic cultures due to the predominance of aerobic bacterial aetiology. While chest x‐rays are frequently insufficient for detecting significant parenchymal changes, contrast‐enhanced chest CT scans remain the most sensitive diagnostic modality [2, 4, 5, 8]. In the present case, the previous CT findings suggested a lung tumour, but the pathological results from the third biopsy revealed no evidence of malignancy. Furthermore, the patient's condition improved clinically and radiologically following the administration of intravenous empirical antibiotics. A multidisciplinary team (MDT) reviewed the previous CT and pathological findings to avoid misdiagnosis. The MDT concluded that the findings indicated necrotizing pneumonia caused by non‐ESBL‐producing *K. pneumoniae* , a fairly common aetiology in this disease, as discussed previously.

Carcinoembryonic antigen (CEA) is a foetal glycoprotein that is elevated in several pathologies. Elevation in CEA level is a well‐known prognostic serologic marker for colorectal cancer (CRC) and is associated with adenocarcinoma of the pancreas, lungs, prostate, ovaries and breast. Multiple studies have reported its relationship with noncancerous, chronic inflammatory conditions such as chronic obstructive pulmonary disease (COPD), obesity, ageing and cigarette smoking. In addition, CEA may be involved in chronic subclinical inflammation. A high CEA level could be regarded as not only a cancer marker but also an inflammation marker that can be increased in chronic inflammation diseases [10]. If the increase in CEA levels exceeds 20 ng/mL and is persistent, the suspicion of malignancy should be strongly considered [11]. In the present case, CEA level reached 8.94 ng/mL, slightly above the normal threshold (typically < 5 ng/mL for non‐smokers). This value remains within the range that can occur in severe pulmonary infections such as necrotizing pneumonia, particularly due to severe inflammation and tissue damage in the lungs, a history of smoking (even though the patient quit smoking 12 years ago) and tissue necrosis and suppuration mimicking malignancy on imaging. Therefore, the elevated CEA in this patient more likely reflects an intense inflammatory response and tissue injury due to infection, rather than an indicator of malignancy. In this patient, the elevated CEA level was attributable to severe inflammation caused by necrotizing pneumonia, not cancer. The diagnosis of necrotizing pneumonia was established based on a combination of clinical presentation, radiological findings, microbiological evidence and histopathological results—along with a positive response to antibiotic therapy, which is inconsistent with lung malignancy.

Treatment for NP requires a multidisciplinary approach. Antibiotic recommendation for NP was Ampicillin‐Sulbactam + Macrolid (first‐line antibiotics) for community‐acquired pneumonia (CAP) and Piperacillin‐Tazobactam or Carbapenem for hospital‐acquired pneumonia (HAP). When risk factors for Methicillin‐resistant *Staphylococcus aureus* (MRSA) are present, it was recommended to add Linezolid or Vancomycin for CAP and add Vancomycin or Linezolid for HAP [12]. Empiric broad‐spectrum antibiotics should be initiated within 4 h of hospital admission, guided by local pathogen patterns and resistance profiles [2]. In severe cases or when complications arise, surgical intervention may be necessary, especially for issues such as massive haemoptysis, empyema, or gangrene [8]. The mortality rate for NP among hospitalised patients within 30 days ranges from 5% to 15%; however, the prognosis is generally favourable if appropriate therapy is administered [2]. Most patients show improvement in respiratory symptoms and achieve complete or minimal fibrotic resolution on radiologic follow‐up within 2–6 months following treatment [6].

In conclusion, this case highlighted the importance of accurate and timely diagnosis in managing necrotizing pneumonia, particularly when it mimics malignancy. Early recognition, supported by appropriate diagnostic tools and targeted antibiotic therapy, is crucial for appropriate management of necrotizing pneumonia and achieving positive outcomes. A multidisciplinary approach is essential to ensure comprehensive care, prevent misdiagnosis and avoid unnecessary treatments. Prompt and precise intervention can significantly enhance patient prognosis and reduce the risk of complications associated with necrotizing pneumonia.

## Author Contributions

**Rahel Yuana Sadikim:** patient evaluation, data collection, manuscript drafting. **Isnin Anang Marhana:** image selection, writing, review and editing, final approval of the manuscript. **Herley Windo Setiawan:** references, writing, review and editing, final approval of the manuscript.

## Ethics Statement

The authors declare that written informed consent was obtained for the publication of this manuscript and accompanying images using the consent form provided by the Journal.

## Conflicts of Interest

The authors declare no conflicts of interest.

## Data Availability

The data that support the findings of this study are available on request from the corresponding author. The data are not publicly available due to privacy or ethical restrictions.
